# Investigating the Formation of In Vitro Immunogenic Gluten Peptides after Covalent Modification of Their Structure with Green Tea Phenolic Compounds under Alkaline Conditions

**DOI:** 10.1021/acs.jafc.4c00334

**Published:** 2024-06-05

**Authors:** Merve Aksoy, Aytül Hamzalıoğlu, Vural Gökmen

**Affiliations:** Food Quality and Safety (FoQuS) Research Group, Department of Food Engineering, Hacettepe University, Beytepe 06800, Ankara, Turkey

**Keywords:** immunogenic gluten peptide, 33-mer, protein–phenol interaction, green tea extract

## Abstract

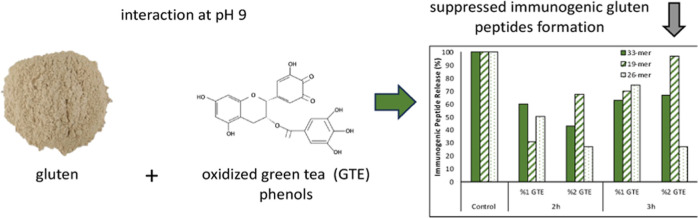

Celiac disease is an autoimmune disorder triggered by immunogenic gluten peptides produced during gastrointestinal digestion. To prevent the production of immunogenic gluten peptides, the stimulation of covalent-type protein–polyphenol interactions may be promising. In this study, gluten interacted with green tea extract (GTE) at pH 9 to promote the covalent-type gluten–polyphenol interactions, and the number of immunogenic gluten peptides, 19-mer, 26-mer, and 33-mer, was monitored after *in vitro* digestion. Treatment of gluten with GTE provided an increased antioxidant capacity, decreased amino group content, and increased thermal properties. More importantly, there was a remarkable (up to 73%) elimination of immunogenic gluten peptide release after the treatment of gluten with 2% GTE at 50 °C and pH 9 for 2 h. All of these confirmed that gluten was efficiently modified by GTE polyphenols under the stated conditions. These findings are important in developing new strategies for the development of gluten-free or low-gluten food products with reduced immunogenicity.

## 1 Introduction

Celiac disease (CD) is an autoimmune enteropathy that is triggered by the ingestion of gluten, a protein found in wheat, barley, and rye, in genetically susceptible individuals. Being rich in glutamine and proline makes gluten resistant to digestive enzymes, resulting in the formation of longer peptide fragments. These fragments stimulate immune reactions, which is why they are called “immunogenic gluten peptides”. These gluten peptides harm the intestinal structure causing villous atrophy due to both innate and adaptive immune reactions following their translocation through the intestinal epithelial membrane.^1^ 33-mer is one of the most immunodominant gluten peptides. CD is important as it causes many clinical manifestations and has a prevalence of 1% of the general population. The only current treatment of CD is the gluten-free diet, which is expensive and difficult to maintain; however, there is a need for safe and efficient new treatments as an alternative to the gluten-free diet.

Phenolic compounds are secondary metabolites of plants and are characterized by one or more hydroxyl groups attached to one or more aromatic rings. Thanks to their ability to scavenge free radicals, phenolic compounds are associated with antioxidant, anticancer, and antidiabetic effects.^2^ On the other hand, phenolic compounds are shown to interact with proteins which results in modified physicochemical attributes of both reacting molecules.^3,4^ These interactions might take place in the food system as well as in the gastrointestinal tract, while leading to inhibition of digestive enzyme activity in the digestive tract. Also, protein–polyphenol interactions provide astringent sensation during oral processing.^4^ The interaction of proteins with polyphenols might be via noncovalent or covalent interactions. The noncovalent interactions take place via reversible weak forces, such as hydrogen bonding, hydrophobic interactions, and ionic bonds, while the covalent interactions are characterized by the formation of strong irreversible bonds between both molecules.^5^ The covalent interaction between proteins and polyphenols can be achieved by enzymatic or nonenzymatic procedures. While the alkaline reaction and free-radical grafting are two commonly used methods for nonenzymatic procedures, enzymatic procedures are based on the presence of polyphenol oxidases.^5^ The alkaline method is a simple but effective method for binding polyphenols to the protein structures and to obtain protein–polyphenol complexes.^4^ This method is based on the oxidation of polyphenols and, subsequently, the formation of quinones. These highly reactive electrophilic intermediate products (quinones) can react with the nucleophilic side chains (e.g., lysine, cysteine, methionine, and tryptophan) of protein.^3,6^ On the other hand, alkaline treatment is a method used in the food industry in many processes such as the production of table olives,^7^ masa, corn flour, tortilla,^8^ alkaline fermentations of raw material with high protein content such as legumes, oilseeds, and fish.^9^ Therefore, alkaline treatment can be adapted to the stimulation of covalent-type interactions of gluten and green tea extract (GTE) polyphenols.

The structure of the protein as well as its functional characteristics, such as digestibility, thermal stability, and antioxidant capacity, are altered as a result of the binding of phenolic compounds to proteins through both covalent and noncovalent interactions.^4,10^ Previous reports demonstrated that the proline residues, which are abundant in gluten, might be potential binding sites for the phenolic compounds.^11^ Recent model studies have demonstrated that phenolic compounds can modify gluten, gliadin, and immunogenic gluten peptides.^12−14^ From this point of view, binding of polyphenols to proline residues of immunogenic gluten peptides can be a promising method to hamper gluten digestion and the release of immunogenic fragments or prevent recognition of gluten peptides by human immune systems (by HLA-DQ2/8 receptors or T-cells etc.).^12^ In the study conducted with a model system composed of epigallocatechin gallate (EGCG) and immunogenic 33-mer peptide, the interaction of EGCG with 33-mer peptide under physiological conditions (at 37 °C and pH 2.0, 6.8 and 7.5) was driven by nonspecific binding, resulting in the formation of polydisperse and EGCG/33-mer complexes causing changes in the peptide structure.^14^ In addition, in another study, molecular dynamics simulations revealed that the interaction of EGCG with 32-mer at room temperature occurs through the different regions of the peptide, particularly in the regions that have more leucine, proline, and glutamine residues.^15^ On the other hand, wheat-derived peptide fractions formed noncovalent complexes with procyanidin B3 which highlights the potential beneficial effects of food polyphenols as a nutritional approach in the modulation of CD.^16^ Furthermore, the addition of GTE to the *in vitro* digestion of gluten reduced the gliadin-mediated intestinal permeability which is assessed by transepithelial electrical resistance of caco-2 cells.^17^ To date, studies investigating the effects of the protein–polyphenol interaction on immunogenic gluten peptides have not focused on covalent-type interactions. Therefore, this study aimed to stimulate the covalent-type interactions of gluten with phenolic compounds under alkaline conditions and to monitor the effects of these interactions on *in vitro* immunogenic gluten peptide release. As a rich and easily accessible source of phenol compounds, GTE was used in this study.

## 2 Materials and Methods

### 2.1 Chemicals

Salts for the preparation of buffers and digestive fluids, including ammonium bicarbonate, sodium bicarbonate, potassium chloride, magnesium chloride, and sodium carbonate, were purchased from Sigma-Aldrich Chemie (Steinheim, Germany). Sodium hydroxide, calcium chloride was purchased from Merck (Darmstadt, Germany). Reagents such as 2,2-diphenyl-1-picrylhydrazyl (DPPH), 6-hydroxy-2,5,7,8-tetramethyl-chroman-2 carboxylic acid (Trolox) (97%), 5,5′-dithiobis (2-nitrobenzoic acid) (DTNB) (98%),4-dithiothreitol (DTT) (97%), and *o*-phthalaldehyde (OPA) were also purchased from Sigma-Aldrich Chemie (Steinheim, Germany). Formic acid (98%) was purchased from J. T. Baker (Deventer, The Netherlands). Cellulose, sodium tetraborate decahydrate, ethyl alcohol (96%), sodium dodecyl sulfate (SDS) (98.5%), tris(hydroxymethyl)aminomethane (TRIS) (99.9%), urea (99.0–100.5%), hydrochloric acid, methanol, acetonitrile, l-serine, l-cysteine, and amino acid standard mix solution (2.5 mM each) were purchased from Sigma-Aldrich Chemie (Steinheim, Germany). The digestive enzymes including α-amylase (≥10 U/mg solid) from porcine pancreas, pepsin (≥250 U/mg solid) from porcine gastric mucosa, pancreatin (4 × USP) from porcine pancreas, and bile extract were also purchased from Sigma-Aldrich (Deisenhofer, Germany). ZIC-HILIC (150 × 4.6 mm, 3.5 μm) columns were purchased from MerckSeQuant (Darmstadt, Germany). Nylon syringe filters (0.45 μm) were obtained from IsoLab (İstanbul, Turkey). Sep-Pak Accell Plus QMA 1 cc Vac cartridges were purchased from Waters (Milford, MA). Aluminum lids and pans were purchased from TA Instruments (New Castle, USA). The immunogenic gluten peptides, 33-mer and 25-mer, were purchased from Elabscience (Texas, USA). Deionized water (5.6 μS/m) was used throughout the analysis and sample preparation. GTE and commercial wheat gluten were purchased from a local market in Ankara.

### 2.2 Preparation of Gluten Samples Treated with GTE

The treatment of gluten (30 g/L) with GTE was applied under the following conditions; 1% (300 mg/L) and 2% (600 mg/L) GTE according to gluten weight, at pH 9 and 50 °C, for 2 and 3 h. Sodium carbonate-bicarbonate buffer (0.1 M) which consists of 3.88 g of sodium bicarbonate and 5.71 g of sodium carbonate per 1 L was used to adjust the pH to 9 in GTE solutions.^18^ Gluten–GTE interactions were carried out by mixing gluten with GTE continuously for 2 or 3 h by using a magnetic stirrer, keeping the temperature at 50 °C, and free from exposure to air. The reason for interactions at 50 °C was to pronounce the complexation of gluten with GTE polyphenols, as investigated by a previous study.^19^ The treatments of cereal bran with a green tea infusion were examined in this work, and the findings indicated that the interaction between amino residues and green tea phenolic compounds was more prominent at higher temperatures. The highest modification of amino residues was at 50 °C, indicating its more pronouncing effect. After treatments, for the isolation of gluten–polyphenol complexes, samples were washed with excess (300 mL) water to remove the excess GTE. To achieve the total removal of excess GTE, the presence of GTE polyphenols was monitored by doing total antioxidant capacity (TAC) analysis in the supernatants after each washing step. Finally, isolation of gluten–phenol complexes could be achieved at the end of a 10-step washing. Then, isolated gluten–polyphenol complexes were lyophilized and kept at 4 °C for further analysis. All of the treatments of gluten with GTE were replicated two times.

### 2.3 Measurement of TAC

#### 2.3.1 TAC Analysis of Gluten Samples by QUENCHER

TAC of modified and native gluten samples was measured using DPPH^*+^ radical solution by the previously established QUENCHER method.^20^

#### 2.3.2 TAC Analysis of Digested Gluten Samples

DPPH assay was applied to supernatants collected from *in vitro* digestion. TAC of the supernatants was measured using a DPPH^*+^ radical solution. To achieve this, 0.2 mL of supernatant from the digestion process was mixed with 1 mL of DPPH solution, and a discoloration (radical quenching) reaction was performed for 3 min during centrifuging at 8000*g*. Optically clear supernatants were placed into cuvettes following centrifugation, and the absorbances were measured at 525 nm using an UV–visible spectrophotometer. The results were expressed in g Trolox Equivalent (TE)/g protein, and the calibration curve was built using Trolox with a concentration range of 100–600 ppm.

### 2.4 Analysis of the Amino Content

Native and GTE-treated gluten samples were treated with 8 M urea before the analysis of the amino and thiol content. Amino content analysis was conducted according to the previously established procedure.^21^ In this procedure, 0.4 mL of serine standard/blank/sample was combined with 3 mL of the OPA reagent, and the mixture was stirred for 5 s. The absorbance value was measured at 340 nm using a UV–visible spectrophotometer (Shimadzu Corp., Kyoto, Japan) after the mixture was left to stand for precisely 2 min. The results were expressed as g of serine equivalent (SE)/g of the sample and were calculated against a serine standard curve.

### 2.5 Analysis of the Total Thiol Content

The thiol content of native gluten and GTE-treated gluten samples which were treated with 8 M urea was determined by derivatization with the previously established DTNB (Elman’s reagent) procedure.^22^

### 2.6 Thermal Analysis

The denaturation temperature of native gluten and GTE-treated gluten samples was measured by using differential scanning calorimetry (DSC) (TA Instruments, New Castle, USA). After 1–3 mg of samples were weighed into an aluminum pan, the aluminum lid and pan were hermetically sealed. Hermetically sealed empty aluminum lids and pans were also used as the references. The thermogram was recorded between 25 and 200 °C with a 10 °C/min heating rate under a dry nitrogen atmosphere with a 30 mL/min flow rate.

### 2.7 In Vitro Digestion of Gluten

*In vitro* peptic and pancreatic digestion for gluten samples: *In vitro* peptic and pancreatic digestion were performed according to a previously established procedure.^23^ 5 mL portion of 10 mM HCl was added to 250 mg of native and GTE-treated gluten samples and incubated at 37 °C for 30 min. Following the incubation, for the simulated gastric phase, 125 μL of pepsin (0.1 mg/mL, 10 mM HCl) was added and incubated at 37 °C for 2 h. After the gastric phase, 410 μL of 1.43 M sodium bicarbonate was added to obtain pH 7.5 and stop the gastric digestion. Then, 75 μL of 50 mg of pancreatin/mL pancreatin buffer which consisted of 5 mL of 10 mM HCl and 410 μL of 1.43 M sodium bicarbonate buffer was added, and it was incubated at 37 °C for 2 h for the intestinal phase. At the end of the intestinal phase, digested samples were immediately cooled in an ice bath, tubes were centrifuged at 8000*g* for 3 min, and supernatants were transferred to another tube and stored at −18 °C for further analysis. *In vitro* digestion experiments were carried out in two replicates.

### 2.8 Determination of Degree of Hydrolysis

After the native and GTE-treated gluten samples were subjected to *in vitro* digestion, the degree of hydrolysis (DH) analysis was performed using the OPA technique^21^ on the supernatants that were collected at the end of the digestion.

### 2.9 Analysis of Free Amino Acids

Aliquots collected from *in vitro* digestion of gluten samples were centrifuged and filtered through a 0.45 μm syringe filter into an autosampler vial. Free amino acid content was analyzed by Waters Acquity TQD LC/MS–MS (Waters, USA). Chromatographic separation was performed on a ZIC-HILIC column (150 × 4.6 mm i.d., 3.5 μm) according to procedure reported by Salman et al.^24^

### 2.10 Analysis of Immunogenic Gluten Peptides in Digested Gluten Samples

Aliquots collected at the end of the intestinal phase were centrifuged, and the cleanup procedure was applied by the solid phase extraction method using a Sep-Pak Accell Plus QMA 1 cc Vac Cartridge. For this, 250 μL of digested samples, 740 μL of water with 0.1% formic acid, and 10 μL of internal standard were added into a tube and then centrifuged at 8000*g* for 3 min. The cartridge was preconditioned with 1 mL of methanol and then 1 mL of deionized water; 1 mL of the supernatant was loaded onto a preconditioned cartridge. Following the washing of the cartridge with 1 mL of water, the sample was eluted with 1 mL of acetonitrile. The eluted sample was evaporated under nitrogen until dryness, and the residue was dissolved in 500 μL of water in an autosampler vial. Immunogenic gluten peptides were analyzed by a Waters Acquity TQD LC/MS–MS (Waters, USA). Chromatographic separation was performed on a ZIC-HILIC column (150 × 4.6 mm i.d., 3.5 μm) by using a gradient elution of 0.1% formic acid in water (A) and 0.1% formic acid in acetonitrile (B) at a flow rate of 1 mL/min at 30 °C. The gradient program for mobile phase B was: 0–4 min 80%, 4–8 min 80 to 40%, 8–12 min 40% to 20%, 12–14 min held at 20%, 14–16 min from 20% to 40%, and 16–18 min from 40% to 80%, held for 4 min. The total chromatographic run time was 22 min. The injection volume was 10 μL. The electrospray source had the following settings: capillary voltage of 2.97 kV; cone voltage of 25 V; extractor voltage of 3 V; source temperature of 130 °C; desolvation temperature of 350 °C; desolvation gas (N_2_) flow of 550 L/h; and cone gas (N_2_) flow of 50 L/h. Peptides were identified by multiple reaction monitoring (MRM) using the parameters given in Table 1. 25 amino acid long peptide having a sequence (PQLPQFLQPQPYPQPQLPYPQPQPF) was used as the internal standard. A calibration curve having 33-mer concentration at a range between 2 and 10 ppm was built. 19-mer and 26-mer were partially quantified according to 33-mer.

**Table 1:** Amino Acid Sequences and MRM Parameters of Immunogenic Gluten Peptides

peptide	amino acid sequence	molecular weight (kDa)	precursor ion *m*/*z* (charge state)	fragmented ions *m*/*z*	retention time (min)
19-mer	LQLQPFPQPQLPYPQPQPF	2263	755.068 (+3)	488.251	8.34
26-mer	LQLQPFPQPQLPYPQPQLPYPQPQPF	3087	1029.543 (+3)	263.139	8.84
33-mer	LQLQPFPQPQLPYPQPQLPYPQPQLPYPQPQPF	3912	979 (+4)	225.4	8.22

### 2.11 Statistical Analysis

Data were statistically analyzed by ANOVA and ANCOVA methods with 95% significance applied using statistic software XLStat (Lumivero, France). The significance of differences between samples was analyzed by the Duncan test (HSD). Differences at *p* < 0.05 were considered significant.

## 3 Results and Discussion

### 3.1 Characterization of Gluten Treated by GTE

Gluten was treated with GTE under certain conditions (pH 9, 50 °C, and for 2 and 3 h). Characterization of gluten treated with GTE was carried out by the analysis of TAC, free amino content, thiol content, and thermal properties.

**Figure 1:**
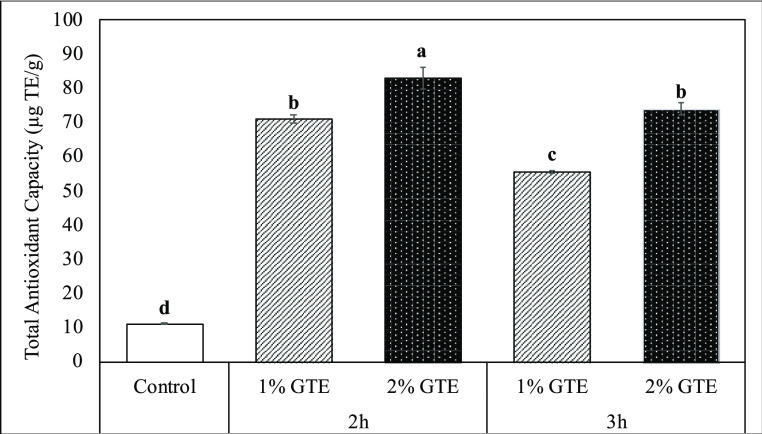
Total antioxidant capacities (μg TE/g) of native (control) and GTE-treated gluten samples.

The hydroxyl group of polyphenols that is attached to the benzene ring maintains their antioxidant function when they are incorporated into proteins, providing higher antioxidant activity. In the case of the incorporation of the phenolic compounds into proteins, their remaining hydroxyl groups exhibit antioxidant function, resulting in the increased antioxidant capacity of proteins.^25^ It is a common practice to evaluate the changes in TAC in order to monitor the proteins’ enhanced antioxidant properties for understanding the interactions.^19^ As given in Figure 1, after its interaction with different concentrations of GTE (1 and 2%) under the specified conditions, gluten exerted more TAC (up to 7.36-fold) than its native form. Analysis of covariance (ANCOVA) was applied to the date, to understand the effect of GTE concentration and interaction time parameters on TAC. According to ANCOVA analysis, correlation coefficients of the effects of GTE concentration and interaction time on total antioxidant capacity were found to be 0.691 and 0.033, respectively (*p* < 0.05). Given the treatment parameters, a much greater correlation coefficient (0.691) confirms that the concentration of GTE had a more noticeable impact on the TAC of the gluten samples treated with GTE. An increase in the GTE concentration allowed better binding of phenolic compounds to gluten structure, resulting in higher TAC. On the other hand, prolonging the exposure time of gluten to GTE did not provide much increase in total antioxidant activity even causing a decrease (*p* < 0.05). In the study of Rohn et al.,^26^ an increasing amount of proteins during the interaction between bovine serum albumin (BSA) and quercetin at pH 9 resulted in lower antioxidative ability of BSA–quercetin complexes. This result is attributed to a second oxidation of BSA–quercetin quinone complexes, which results in the formation of protein–quercetin–protein complexes. The protein–quercetin–protein reactions in which reactive quercetin sites are involved are partly responsible for the loss of antioxidative ability. Therefore, in our study, prolonging the treatment duration of gluten with GTE from 2 to 3 h might possibly lead to a second oxidation of gluten–quinone complexes and the formation of gluten–quinone–gluten cross-link groups which result in lower antioxidant activity.

As the residual amino groups are one of the target sites for protein–phenol interaction, a decrease in the number of free amino groups might be an indicator to verify these interactions. The total amino content of native gluten, as indicated in Table 2, was determined to be 178.91 (3.28) g SE/g sample, while it diminished by 17.46% after being treated with 2% GTE at pH 9 for 2 h. Moreover, an increase in the GTE concentration resulted in less free amino group content, which indicates more incorporation of GTE phenolic compounds through the amino side groups of gluten. On the other hand, prolonging treatment time did not cause a reduction in the amino group content at the same GTE concentration. These results may indicate the modification of gluten through amino groups as a result of covalent interactions because the samples were treated with 8 M urea before the analysis, which most likely destroyed noncovalent interactions.

**Table 2:** Changes in Thiol Group (μmol Cys/g), Amino Group (μg/g), and Melting Point (*T*_m_, °C) in Native and GTE-Treated Gluten Samples^a^

interaction conditions		results		
GTE concentration	time	thiol group content (μmol Cys/g)	amino group content (μg SE/g)	*T*_m_ (°C)
control		36.35 ± 2.56^a^	178.91 ± 4.64^a^	82.7
%1 GTE	2 h	35.90 ± 0.55^a^	159.18 ± 2.93^b^	93.16*
%2 GTE	2 h	36.48 ± 1.10^a^	147.67 ± 0.66^c^	84.00*
%1 GTE	3 h	36.29 ± 0.09^a^	154.84 ± 0.22^b,c^	93.45*
%2 GTE	3 h	37.26 ± 2.48^a^	148.44 ± 6.19^c^	84.58*

a Data were expressed as mean ± standard deviation. SE corresponds to serine equivalent. The values followed by the same lowercase letters are not statistically different within a row (*p* > 0.05). * indicates a statistically significant difference according to the *t*-test (*p* < 0.05).

Another target residue for oxidized phenolic compounds (quinones) for interacting with proteins are thiol groups of proteins; therefore, the loss in the total thiol content could be an indicator of interactions in the samples treated with GTE. As given in Table 2, the treatment of gluten with different concentrations of GTE for both 2 and 3 h did not show a significant decrease in the thiol group content (*p* > 0.05). However, in a study investigating the interactions of flax seed proteins and hydroxytyrosol at pH 9 for 2 h, the thiol group in flax seed protein decreased from 40.39 ± 1.30 to 2.02 ± 0.37 nmol/mg protein due to the covalent modification by hydroxytyrosol.^27^ There are two proposed mechanisms for interactions between dimethyl trisulfide and Cys–Cys residues in β-lactoglobulin (β-LG) protein after the reaction with the free cysteine group; (i) reduction of disulfide bonds and binding of dimethyl trisulfide through these exposed thiol groups in β-LG and (ii) binding of dimethyl trisulfide through free thiol side groups in β-LG.^28^ Moreover, it was reported that the persimmon tannins caused a reduction of S–S in gluten; however, the reduction of disulfide bonds was not accompanied by an increase in free thiol groups equally.^29^ This result is attributed to the interaction of persimmon tannins and thiol groups in gluten. Therefore, the reason for not observing changes in the total thiol content after the treatment of gluten with GTE polyphenols in our results might be the cleavage of the disulfide bond first and then binding of GTE polyphenols to all corresponding free thiol groups in gluten. On the other hand, considering immunogenic gluten peptide sequences that do not represent any cysteine residues, even if GTE phenolic components were bound via thiol groups in gluten, this may not be effective in the elimination of immunogenic gluten peptides.

The structure, denaturation temperature, enthalpy of unfolding, and heat capacity of proteins are usually altered by their interaction with phenol compounds.^4,30^ These alterations are due to the unfolding of the protein once phenol compounds are attached to them.^3,31^ Therefore, the changes in the thermal stability of proteins might be a method to determine whether their interaction with phenol compounds takes place. The changes in thermal stability of modified gluten samples were monitored by measuring their melting point using DSC. The basis of DSC is the measurement of the thermal power as a function of temperature or time that is needed to keep the reference and sample at the same temperature. The point of equilibrium of the native protein and its denatured conformations is known as the midpoint of the transition or melting temperature (*T*_m_). As an outcome, more stable molecules are defined as molecules or samples with higher *T*_m_ values.^32^ Interaction of proteins with phenolic compounds leads to changes in *T*_m_ values which might be used as an indicator of the interaction.^33,34^ In a study, the interaction of soy protein with phenolic acids at pH 9 for 24 h resulted in the increase of soy protein denaturation temperature from 93 to 99 °C.^35^Table 2 gives *T*_m_ values, derived from the DSC thermograms, for both native gluten and gluten treated with GTE. The *T*_m_ value of native gluten was determined to be 82.7 °C, in agreement with previous findings.^36,37^ On the other hand, the samples of gluten treated with GTE increased *T*_m_ values which varied between 84.00 and 93.45 °C. This increase in *T*_m_ values in this study might be evidence of the interactions between gluten and GTE phenol compounds taking place under specified conditions.

### 3.2 Digestive Characteristics of Gluten Samples

**Figure 2:**
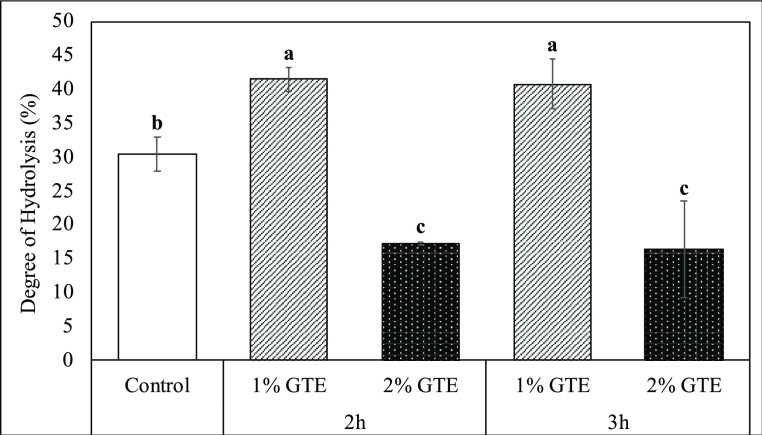
Degree of hydrolysis (%) of native (control) and GTE-treated gluten samples following *in vitro* digestion.

In this work, native and GTE-treated gluten samples were subjected to *in vitro* digestion, and bioaccessible fractions, corresponding to the supernatant obtained by centrifugation at the end of the intestinal phase, were obtained. The degree of protein hydrolysis was measured in bioaccessible fractions to see the effects of protein–polyphenol interactions on gluten digestibility. The determination of the DH is based on the reaction between the amino groups of proteins and the OPA reagent which results in the formation of the colored compound, and the absorbance value of this colored compound is measured. The DH of native gluten and GTE-treated gluten is given in Figure 2. Compared to gluten, there was an increase in the DH of gluten treated with 1% GTE. This might be a result of the unfolding of the high-order structure of gluten because of its interactions with GTE under alkaline conditions. In addition, the incorporation of GTE quinones did not hinder the accessibility of the active sites of digestive enzymes to gluten. However, the degree of gluten hydrolysis decreased when the GTE concentration was less than 2%. As mentioned before (Figure 1), the modification of gluten was more pronounced when it was treated with a higher amount of GTE phenolics. Oxidation of GTE polyphenols and their subsequent binding could be stimulated more in gluten samples treated with 2% GTE, which led to a reduction in the digestibility of gluten through probable blockage of the active sites targeted for digestive enzymes. As the digestive enzymes, trypsin prefers to cleave residues of basic amino acids like arginine and lysine, while chymotrypsin prefers to cleave residues of aromatic amino acids like phenylalanine, tyrosine, and tryptophan.^38^ Therefore, the decrease in these amino acids (Table S2) might support the possible binding of GTE quinones to gluten through the active sites for digestive enzymes. Similar results were also obtained by others, the covalent interaction of soy protein isolate with EGCG at pH 9 resulted in less digestibility.^39^ Moreover, protein digestibility was affected significantly by the EGCG concentration, while it decreased from 76.17 ± 1.56 to 27.87 ± 2.67% with the highest (5 mM) EGCG concentration.

As the protein digestibility is affected by the treatment with GTE, to see the effect of the GTE–gluten interaction on the bioaccessibility of amino acids is also of importance. For this purpose, following the *in vitro* digestion process, the free amino acid contents of both native and GTE-treated gluten samples were measured. Changes in the amounts of released individual amino acids, total free amino acids, essential amino acids, and total reactive amino acids after *in vitro* digestion of native and gluten treated with GTE are given in the Supporting Information (Tables S1 and S2). The total bioaccessible amino acid content was found to be 55.85 ± 1.53 mg/g in native gluten. Also, there was no significant reduction found in the total bioaccessible and essential amino acid content due to the treatment of gluten with GTE which indicated that the bioaccessibility of amino acids was not adversely affected by the gluten–GTE interaction. The production of peptides and nonpeptide compounds, gene expression regulation, cell signaling pathways, energy and nutrition metabolism, and immunological function are all impacted by essential amino acids.^40^ Therefore, considering these functions of essential amino acids, these findings were notable, in that the gluten–GTE interaction did not alter the bioaccessibility of essential amino acids.

Lysine, asparagine, tyrosine, methionine, histidine, tryptophan, and arginine amino acids have chemically reactive side groups^41^ that possibly interact with GTE phenolic compounds and might be referred to as reactive amino acids. p*K*_a_ values of amino, phenolic, imidazole, and guanidyl groups are 10.2, 9.6, 7.0, and 13.8, respectively.^42^ The interaction between electrophilic quinones and amino acid side chains might be favorable at pH 9 because of the p*K*_a_ values of the side chains of the amino acids. However, the released reactive amino acid content of native gluten after *in vitro* digestion was found to be 18.15 ± 0.02 mg/g, it has been decreased significantly (*p* < 0.05) due to the treatment of gluten with 2% GTE for 2 and 3 h. This result suggested that the interaction between quinones and side chains of amino acids took place under these conditions. However, the total reactive amino acid content of gluten samples increased after being treated with 1% of GTE at pH 9. This result indicated that the GTE concentration was also a determinant for binding.

Besides these amino acids, the proline residues and/or proline repeats are one of the factors of protein–phenol interactions.^38^ Furthermore, it has been demonstrated in numerous studies that immunogenic peptides, proline-rich proteins such as salivary protein, and casein, interact with various phenolic compounds.^11,17,43^ The released proline content of native gluten following *in vitro* digestion was found as 0.56 ± 0.04 mg/g; however, the proline content of GTE-treated gluten was not detected. The proline residues may therefore additionally act as preferred binding sites, as evidenced by the decline in the proline content of gluten following the treatment with GTE under all interaction conditions.

**Figure 3:**
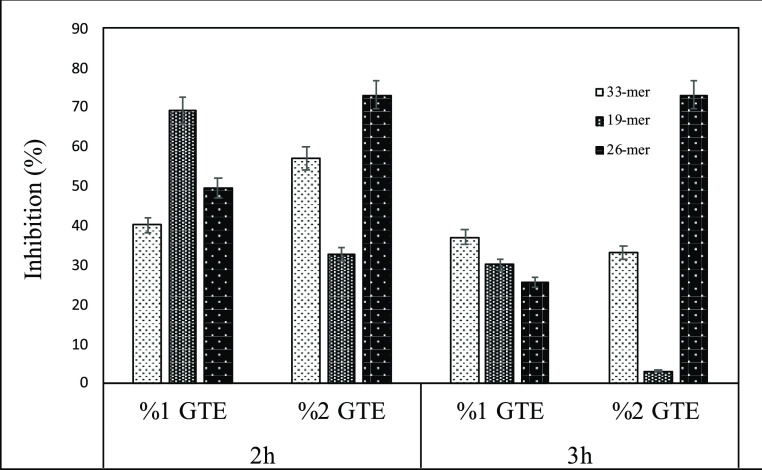
Percentage inhibition of immunogenic peptides of GTE-treated gluten samples subjected to *in vitro* digestion.

Due to the high proline and glutamine content, partial digestion of gluten results in the formation of immunogenic peptides, which trigger pathogenesis of CD. For this reason, as the main purpose of this study, changes in immunogenic peptides were monitored to understand the effect of treatment of gluten with GTE at pH 9 on the release of immunogenic peptides. Six immunogenic peptides (including 13-, 19-, 26-mer, and 33-mer) were quantified in the bread after *in vitro* digestion.^44^ However, in our samples, the presence of 33-mer, 26-mer, and 19-mer was confirmed. The concentration of the 33-mer peptide of gluten subjected to *in vitro* digestion was found as 4.84 ± 0.25 mg/g gluten. This result is consistent with the study that screened the 33-mer concentrations in 38 different wheat flour where 33-mer concentrations varied between 0.09 and 0.60 mg/g flour after enzymatic hydrolysis.^45^ As given in Figure 3, the treatment of gluten with GTE phenolic compounds provided the inhibition of gluten peptides in the range of 3–73%. These findings indicated that the modifications of gluten, which are most probably covalent modifications at pH 9, provided less immunogenic peptide release during digestion. In the formation of most immunogenic gluten peptide, 33-mer, the highest decrease (57%) was provided by the treatment with %2 GTE for 2 h. However, immunogenic gluten peptide release from gluten treated with 1% GTE was much more than from gluten treated with 2% GTE. The sequences of the gluten peptides represent potential binding sites for the phenolic compounds, such as aromatic side chains of tyrosine and phenylalanine and hydrophobic sections of proline, leucine, and glutamine.^14^ It was found that EGCG interacted with 32-mer at room temperature through the different regions of the peptide, especially those with more leucine, proline, and glutamine residues.^15^

Accordingly in this study, the reduction in immunogenic peptide release from gluten treated with GTE phenolic compounds might be related to these amino acids. The changes in amino acids released during digestion, as given in Supporting Information (Table S1), reduction in tyrosine, and glutamine, present in immunogenic gluten peptide sequences were observed. These results might indicate that the binding of GTE phenol compounds to gluten occurred through these amino acids during treatment with 2% GTE. On the other hand, there was no significant reduction in these amino acids in gluten samples treated with 1% GTE. This is also consistent with the results of the degree of protein hydrolysis. However, inhibition of immunogenic gluten peptides was provided by the treatment of gluten with 1% GTE, possibly as a result of its fragmentation into shorter peptides.

In this study, the interaction of gluten and GTE under alkaline conditions was studied in terms of immunogenic gluten peptides for the first time. The covalent-type interactions of gluten with GTE phenols efficiently occurred at pH 9, 50 °C as it was supported by the changes in the TAC, the amount of amino and thiol groups, and the thermal characteristics of the gluten. Oxidation of GTE phenolics under alkaline conditions and their subsequent binding to gluten could take place through the amino groups of gluten and provide increased antioxidant capacity. Furthermore, those modifications had an effect on the digestive characteristics of gluten. Covalent-type interaction of gluten with oxidized GTE polyphenols reduced its digestibility; however, there was no decrease in total free and essential amino acids, indicating that this treatment did not affect the bioaccessibility of amino acids. Moreover, less immunogenic gluten peptide was formed after *in vitro* digestion of gluten treated with GTE under alkaline conditions. The treatment of gluten with 2% GTE at pH 9 for 2 h provided the highest inhibition of (57%) of the 33-mer peptide, which is known as the most immunogenic gluten peptide. These findings demonstrated that the production of less immunogenic gluten ingredients and products can be achieved by covalent-type protein–polyphenol interactions. The results of this study are important for the development of wheat-based, easily accessible, and cheap gluten ingredients for those suffering from CD or nonceliac gluten sensitivity. The conditions tested in this study can easily be adapted to pilot scale or large scale; however, they might affect the functional and technological properties of gluten, which should be assessed in bread-making or other various food processes. To avoid possible alterations in technofunctional properties, less destructive covalent-type protein–phenol interactions might be promoted by using enzymatic processes.

## Supporting Information Available

Changes in amino acid contents of native and GTE-treated gluten samples after in vitro digestion (PDF)
