# Supplementary material for: Investigating the Formation of In Vitro Immunogenic Gluten Peptides after Covalent Modification of Their Structure with Green Tea Phenolic Compounds under Alkaline Conditions
Source: J Agric Food Chem. 2024 Jun 5;72(24):13898–905. doi: 10.1021/acs.jafc.4c00334 (PMC11191684; doi:10.1021/acs.jafc.4c00334)
Supplement: Supplementary file 1 — jf4c00334_si_001.pdf [file jf4c00334_si_001.pdf]

Supporting Information

Investigating the formation of in vitro immunogenic gluten peptides after covalent modification of its structure with green tea phenolic compounds under alkaline conditions

Merve Aksoy[a], Aytül Hamzalıoğlu[a*], Vural Gökmen[a]

[a]Food Quality and Safety (FoQuS) Research Group, Department of Food Engineering, Hacettepe University, 06800 Beytepe, Ankara, Turkey

*e-mail: aytulhamzalioglu@hacettepe.edu.tr

Table S1. Changes in released amino acid contents (mg/g) of native and GTE-treated gluten samples after *in vitro* digestion.

Interaction Conditions GTE Concentration	Time	PHE	LEU	ILE	MET	VAL	TRP	TYR	THR	ALA	SER	GLN	ASN	HIS	LYS	ARG	PRO
Control		3.68 ± 0.32^{c}	12.48 ± 2.38^{b}	5.89 ± 0.49^{a,b,c}	0.64 ± 0.08^{b}	2.47 ± 0.30^{a,b,c}	2.12 ± 0.10^{a}	2.57 ± 0.02^{b,c}	1.21 ± 0.13^{a}	1.72 ± 0.23^{a,b}	1.20 ± 0.05^{a}	8.50 ± 0.62^{c}	0.58 ± 0.02^{a}	0.80 ± 0.02^{b,c}	8.56 ± 0.45^{c}	2.86 ± 0.18^{a}	0.56 ± 0.04
%1 GTE	2h	7.30 ± 0.20^{a}	23.12 ± 0.92^{a}	9.76 ± 2.82^{a}	1.29 ± 0.44^{a,b}	3.3 ± 0.93^{a,b}	3.16 ± 0.82^{a}	4.18 ± 0.43^{a}	1.28 ± 0.34^{a}	2.19 ± 0.94^{a}	1.34 ± 0.45^{a}	13.79 ± 0.73^{a}	0.66 ± 0.20^{a}	1.58 ± 0.50^{a}	14.05 ± 0.73^{a}	7.09 ± 0.62^{a}	n.d.
%1 GTE	3h	5.37 ± 0.29^{b}	22.93 ± 0.32^{a}	8.84 ± 1.74^{a,b}	1.42 ± 0.33^{a}	3.72 ± 0.85^{a}	2.68 ± 0.24^{a}	3.18 ± 0.01^{b}	1.11 ± 0.20^{a,b}	1.68 ± 0.30^{a,b}	1.04 ± 0.16^{a,b}	12.15 ± 0.06^{b}	0.51 ± 0.01^{a,b}	1.34 ± 0.16^{a,b}	12.18 ± 0.31^{b}	6.49 ± 0.25^{a}	n.d.
%2 GTE	2h	3.12 ± 0.55^{c}	15.45 ± 0.13^{b}	4.59 ± 0.84^{c}	0.63 ± 0.12^{b}	1.53 ± 0.11^{c}	0.90 ± 0.15^{b}	1.95 ± 0.27^{c,d}	0.62 ± 0.05^{b}	0.92 ± 0.11^{b}	0.62 ± 0.09^{b}	6.56 ± 0.70^{d}	0.28 ± 0.01^{c}	0.67 ± 0.08^{c}	6.69 ± 0.72^{d}	3.26 ± 0.38^{b}	n.d.
%2 GTE	3h	3.10 ± 0.52^{c}	16.15 ± 2.03^{b}	5.37 ± 0.21^{b,c}	0.65 ± 0.13^{b}	1.77 ± 0.1^{b,c}	0.93 ± 0.20^{b}	1.69 ± 0.21^{d}	0.68 ± 0.01^{b}	1.17 ± 0.01^{a,b}	0.79 ± 0.11^{a,b}	7.25 ± 0.43^{c,d}	0.33 ± 0.01^{c}	0.77 ± 0.09^{b,c}	7.40 ± 0.44^{c,d}	3.33 ± 0.07^{b}	n.d.

The values followed by the same lowercase letters are not statistically different within a column for each amino acid separately ($p > 0.05$).
Data were expressed as mean ± standard deviation.
n.d: not detected.

Table S2. Changes in the contents (mg/g) of total amino acids, essential amino acids, reactive amino acids and amino acids that digestive enzymes act on in native and GTE-treated gluten samples after *in vitro* digestion.

Interaction Conditions		TOTAL AMINO ACID CONTENT	TOTAL ESSENTIAL AMINO ACID CONTENT	TOTAL REACTIVE AMINO ACID CONTENT	AMINO ACIDS THAT DIGESTIVE ENZYMES ACT ON
GTE Concentration	Time				
Control		55.85 ± 1.53^{b}	34.74 ± 1.77^{c}	18.15 ± 0.02^{c}	19.79 ± 0.17^{c}
%1 GTE	2h	94.08 ± 6.31^{a}	60.25 ± 3.04^{a}	32.01 ± 2.29^{a}	35.78 ± 1.35^{a}
%1 GTE	3h	84.62 ± 4.02^{a}	54.45 ± 2.06^{b}	27.79 ± 0.68^{b}	2.90 ± 0.10^{b}
%2 GTE	2h	47.80 ± 0.48^{b}	32.05 ± 0.31^{c}	14.39 ± 0.56^{d}	15.92 ± 1.31^{d}
%2 GTE	3h	51.37 ± 3.02^{b}	34.39 ± 2.66^{c}	15.10 ± 0.36^{d}	16.45 ± 0.90^{d}

The values followed by the same lowercase letters are not statistically different within a column for each amino acid separately ($p > 0.05$).
Data were expressed as mean ± standard deviation.
